# Dimethyl fumarate eliminates differentially culturable *Mycobacterium tuberculosis* in an intranasal murine model of tuberculosis

**DOI:** 10.3389/fcimb.2022.957287

**Published:** 2022-08-24

**Authors:** Sarah M. Glenn, Obolbek Turapov, Vadim Makarov, Douglas B. Kell, Galina V. Mukamolova

**Affiliations:** ^1^ Division of Biomedical Services, University of Leicester, Leicester, United Kingdom; ^2^ Leicester Tuberculosis Research Group, Department of Respiratory Sciences, University of Leicester, Leicester, United Kingdom; ^3^ Research Center of Biotechnology, Russian Academy of Sciences, Moscow, Russia; ^4^ Department of Biochemistry and Systems Biology, Institute of Systems, Molecular and Integrative Biology, Faculty of Health and Life Sciences, University of Liverpool, Liverpool, United Kingdom; ^5^ The Novo Nordisk Foundation Centre for Biosustainability, Technical University of Denmark, Lyngby, Denmark

**Keywords:** *Mycobacterium tuberculosis*, tuberculosis, differentially culturable bacteria, treatment, persisters, resuscitation-promoting factor, dimethyl fumarate

## Abstract

Tuberculosis (TB) claims nearly 1.5 million lives annually. Current TB treatment requires a combination of several drugs administered for at least 6 months. *Mycobacterium tuberculosis* (Mtb), the causative agent of TB, can persist in infected humans and animals for decades. Moreover, during infection, Mtb produces differentially culturable bacteria (DCB) that do not grow in standard media but can be resuscitated in liquid media supplemented with sterile Mtb culture filtrates or recombinant resuscitation-promoting factors (Rpfs). Here, we demonstrate that, in an intranasal murine model of TB, Mtb DCB are detectable in the lungs after 4 weeks of infection, and their loads remain largely unchanged during a further 8 weeks. Treatment of the infected mice with dimethyl fumarate (DMF), a known drug with immunomodulatory properties, for 8 weeks eliminates Mtb DCB from the lungs and spleens. Standard TB treatment consisting of rifampicin, isoniazid, and pyrazinamide for 8 weeks reduces Mtb loads by nearly four orders of magnitude but does not eradicate DCB. Nevertheless, no DCB can be detected in the lungs and spleens after 8 weeks of treatment with DMF, rifampicin, isoniazid, and pyrazinamide. Our data suggest that addition of approved anti-inflammatory drugs to standard treatment regimens may improve TB treatment and reduce treatment duration.

## Introduction

Tuberculosis (TB) remains a significant global challenge that affects nearly 10 million people annually (WHO, 2021). TB treatment is long and requires a combination of several drugs, which may result in low compliance and rise of multidrug-resistant TB. The effectiveness of treatment is affected by many factors, namely, the extent of pathological changes and cavitation, status of the immune system, chronic non-infectious diseases, and side effects of drugs (Dartois and Rubin, 2022). Cavitary TB, accompanied by high *Mycobacterium tuberculosis* (Mtb) loads in sputum samples, is more challenging to cure and often requires prolonged treatment (Imperial et al., 2018). It becomes increasingly clear that meeting the WHO STOP TB objectives (WHO, 2008) is not possible without the development of personalised treatment and a deep understanding of host–pathogen interactions, physiological adaptation of Mtb to the immune pressures, and molecular mechanisms of Mtb survival during chemotherapy. The concept of persisters, the enigmatic bacilli that are not killed by bactericidal drugs and persist in infected humans and animals for a long period of time, has highlighted the importance of bacterial physiological state in successful eradication of TB (Gold and Nathan, 2017). While the precise nature of these persisters is obscure, it is generally accepted that heterogenous Mtb populations arise during infection (Manina et al., 2015) and can be recovered from infected tissue or TB clinical samples (Mukamolova et al., 2010; Dhillon et al., 2014; Hu et al., 2015; Chengalroyen et al., 2016; Turapov et al., 2016; Rosser et al., 2018; Dusthackeer et al., 2019). These populations differ by staining patterns (Garton et al., 2008; Manina et al., 2015) or by cultivation requirements (Mukamolova et al., 2010; Turapov et al., 2016; Chengalroyen et al., 2016). Simple growth assays revealed at least three distinct Mtb populations in clinical samples: plateable Mtb, producing colonies on solid agar; non-plateable Mtb, recovering in liquid media; resuscitation-promoting factor (Rpf)–dependent or culture supernatant (CSN)–dependent Mtb, growing in the presence of sterile CSN or recombinant Rpf (Mukamolova et al., 2010; Turapov et al., 2016). Our current understanding of Mtb resuscitation of non-plateable and CSN-dependent Mtb is limited; therefore, the term “differentially culturable bacteria” more accurately defines Mtb that cannot grow in standard media and have special cultivation and resuscitation requirements (Chengalroyen et al., 2016).

Regardless of the nature of resuscitating factor, DCB are apparently more resistant to first-line drugs, enriched in treated patients (Mukamolova et al., 2010; Turapov et al., 2016; McAulay et al., 2018; Beltran et al., 2020) and associated with TB relapse in the Cornell mouse model of TB (Hu et al., 2015; Hu et al., 2019).

We have previously proposed that formation of Rpf-dependent mycobacteria is triggered by the specific *in vivo* environment; the host inflammatory response likely plays a critical role in this process (Turapov et al., 2014). Application of anti-inflammatory compounds reduced the duration of TB treatment, indirectly suggesting that the inflammatory response may trigger persister formation (Gold et al., 2012). However, dynamics of DCB or Rpf-dependent Mtb was not investigated in the study. Here, we show that DCB are generated in the murine lungs after 4 weeks of infection *via* an intranasal route. The treatment of the infected mice with dimethyl fumarate (DMF) for 4 weeks removes DCB, suggesting a strategy for controlling DCB burden. DMF is a well-known drug for management of psoriasis and multiple sclerosis (Rommer et al., 2018; Kourakis et al., 2020). This chemical possesses profound anti-inflammatory properties (Seidel and Roth, 2013). In particular, DMF was shown to reduce NF-κB–mediated pro-inflammatory cytokine release in human peripheral blood mononuclear cells and down-regulate the production of nitric oxide synthase and IL-1β, TNF-α, and IL-6 in cultured microglia (reviewed by Mills et al., 2018). DMF promoted post-ischemic recovery of mice and was well tolerated at a concentration of 45 mg/kg (Yao et al., 2016).

DMF can kill Mtb at a concentration of 12.5 mM (Ruecker et al., 2017); however, its effect on Mtb growth at lower concentrations has not been studied. Our pilot results show that DMF has an inhibitory effect on Mtb growth *in vitro* at a concentration of 25 µM but does not impact on loads of plateable Mtb *in vivo*. Nevertheless, it facilitates the removal of DCB in infected murine organs when used on its own or in combination with standard treatment.

## Methods

### Organism and media

The Mtb H37Rv strain was grown in 7H9 Middlebrook’s broth supplemented with 10% (v/v) oleic acid albumin dextrose (OADC) enrichment, 0.2% (v/v) glycerol, and 0.05% (w/v) Tween 80 (referred to as supplemented 7H9 broth or s7H9). Solid 7H10 agar supplemented with OADC was used for the determination of colony-forming units (CFUs). PANTA supplements were added for the prevention of contamination. CSNs were obtained from logarithmic phase cultures (OD580 ~0.8–1.0) as previously described (Turapov et al., 2016). CSN preparations were passed twice through 0.22-µm filters, and sterility of all batches was confirmed by incubation of CSN aliquots at 37°C for 12 weeks. Media and supplements were purchased from DIFCO™; DMF, rifampicin, isoniazid, and pyrazinamide were purchased from Sigma-Aldrich. For preparation of frozen stocks, bacteria were washed and frozen in phosphate-buffered saline (PBS).

### Animal infection experiments

Animal experiments were performed in accordance with the Animals (Scientific Procedures) Act, under project license P7B01C07A. The University of Leicester Ethical Committee and the U.K. Home Office approved the experimental protocols. Six- and eight-week-old female BALB/c mice were bred and maintained in house for the study. Mice were randomised to groups. Mice were lightly anaesthetised with 2.5% (v/v) isoflurane over oxygen (1.8–2 L min^−1^) and infected intranasally with 5 × 10^3^ mycobacteria resuspended in 50 µl of PBS. For each time point, bacterial loads from five to eight separate mice were determined.

### Treatment

After 4 weeks of infection, mice began treatment *via* oral gavage. Formulations were prepared in PEG 400. Four groups of mice included (i) control (PEG 400), (ii) DMF (DMF at 45 mg/kg twice a day as previously described by Yao et al., 2016), (iii) standard treatment RHZ (rifampicin at 10 mg/kg, isoniazid at 25 mg/kg, and pyrazinamide at 150 mg/kg), and (iv) DRHZ (standard treatment RHZ with DMF). Two doses of DMF were given to mice in DMF and DRHZ groups. Mice were treated for up to 8 weeks and Mtb counts determined after 4 and 8 weeks of treatment. Minimum inhibitory concentration of DMF for Mtb growth was determined in s7H9 according to the published protocols (Jorgensen et al., 1999).

### Determination of bacterial counts

Mtb loads in organs were assessed by CFU and most probable number (MPN) count assays (Turapov et al., 2014). Briefly, mice were humanely killed by cervical dislocation and immediately dissected. Lungs or spleens were homogenized in 5 ml of 7H9 medium containing sterile MP Biomedicals™ Lysing Matrix S beads using a FastPrep-24 homogenizer (MP Biomedicals). Homogenates were serially diluted in 7H9 medium and used for CFU and MPN count assays. MPN counts were determined in s7H9 (MPN) or in 7H9 containing 50% (v/v) CSN. MPN counts were calculated using the MPN calculator program (Jarvis et al., 2010). Unpaired *t*-test was used for statistical analysis.

### Key definitions

*DCB_7H9_ Mtb* – differentially culturable bacteria that resuscitate in s7H9 liquid media.

*DCB_CSN_ Mtb* – differentially culturable bacteria that resuscitate in s7H9 liquid media supplemented with sterile CSN.

*DCB Mtb* – all Mtb bacteria that do not grow on solid media but resuscitate in liquid media.

*Plateable Mtb* – bacteria that produce colonies on solid 7H10 agar.

*Resuscitation Index* (RI) – is the ratio of resuscitating Mtb to plateable Mtb. CSN consistently showed the highest resuscitation effect; we therefore determined RI only for CSN. RI = log_10_ (MPN_CSN ml^-1^) - log_10_ (CFU ml^-1^).

## Results

### DCB are produced in murine lungs after 4 weeks of infection

**Figure 1:**
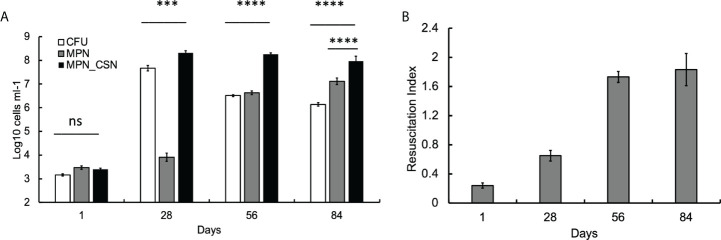
Assessment of Mtb loads in the infected murine lungs over a 12-week infection period. Mice were culled after 1, 28, 56 and 84 days of infection. At 56 and 84 days, mice received PEG (no treatment control). **(A)** CFU, MPN and MPN_CSN counts were determined in the lung homogenates. Data shown as means ± STDV (*N* = 5). **(B)** The resuscitation indices were calculated using the following formula: RI = log_10_ (MPN_CSN ml^-1^) – log_10_ (CFU ml^-1^). Ns, non-significant; *p* > 0.05; ***p < 0.001; ****p < 0.0001 unpaired *t*-test.

We first investigated the dynamics of plateable Mtb and DCB in the control animal group using CFU and MPN count assays. We found that, at 24 h post-infection, CFU, MPN, and MPN_CSN counts were nearly identical (around 3.2 log_10_ bacteria ml^-1^), suggesting that, at the onset of experiment, most bacteria were plateable (**Figure 1A**). As the infection progressed, CFU increased by five orders of magnitude and peaked at 4 weeks of infection; further infection resulted in a substantial decrease in CFU counts, which stabilized at 6 log_10_ CFU ml^-1^ at the end of the experiment (**Figure 1A**). The MPN counts obtained in 7H9 showed an opposite pattern, and after the initial lower increase (presumably due to growth inhibition), they progressively increased throughout the study. MPN_CSN counts remained largely unchanged after 4 weeks of infection and were significantly higher than CFU or MPN counts (*p* < 0.001). At 8 weeks of infection, the resuscitation index reflecting the difference between plateable and CSN-resuscitated Mtb was 1.8 (**Figure 1B**). Our data showed that DCB are produced in the lungs in the intranasal infection model, confirming our previous findings obtained with *Mycobacterium bovis* Bacillus Calmette-Guerin (BCG) (Turapov et al., 2014). Thus, generation of DCB *in vivo* is not a species-specific phenomenon and can be observed in Mtb and *M. bovis* (BCG). Importantly, DCB_CSN_ was the dominating Mtb population recovered from the lungs at 4, 8, and 12 weeks of infection.

### Treatment with the RHZ combination for 8 weeks significantly reduces bacterial loads but does not eliminate DCB

**Figure 2:**
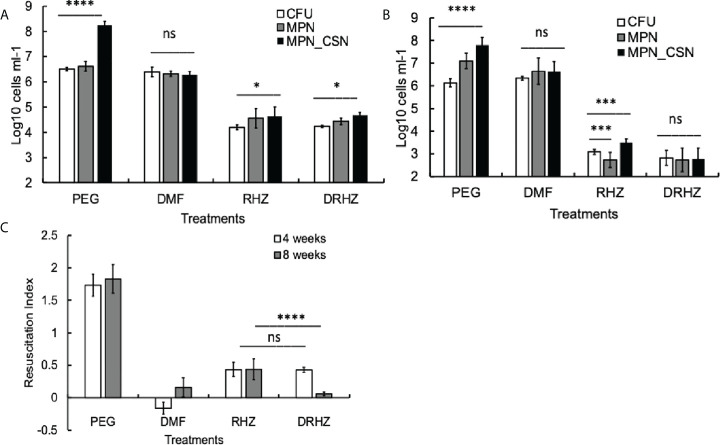
Effect of dimethyl fumarate (DMF), standard treatment and standard treatment with DMF on Mtb loads in the infected murine lungs after 4 **(A)** and 8 weeks of treatment **(B)**. Mice were infected intranasally as described in *Methods* and treatment began after 4 weeks of infection. Murine lung homogenates were used for CFU, MPN and MPN_CSN assays. **(C)** Resuscitation indices. Data shown as means ± STDV (*N* = 7) **(B)**. The resuscitation indices were calculated using the following formula: RI= log_10_ (MPN_CSN ml^-1^) – log_10_ (CFU ml^-1^). PEG, control PEG 400 group; DMF, dimethyl fumarate group; RHZ, rifampicin, isoniazid and pyrazinamide group; and DRHZ, rifampicin, isoniazid, pyrazinamide and dimethyl fumarate group; Ns, non-significant; *p* > 0.05; *p < 0.05; ***p < 0.001; ****p < 0.0001 unpaired *t*-test.

Exposure of mycobacteria to antimicrobials such as rifampicin or isoniazid leads to the accumulation of Rpf-dependent mycobacteria (Loraine et al., 2016). We next investigated whether standard treatment, consisting of rifampicin (R), isoniazid (H), and pyrazinamide (Z), had any effect on Mtb counts in the lungs after 4 and 8 weeks of treatment. Mtb loads of plateable Mtb were reduced by two and three orders of magnitude at 4 and 8 weeks of treatment, respectively (**Figures 2A, B**). Importantly, this treatment reduced but did not completely eliminate DCB_CSN_ as MPN_CSN counts were significantly higher than CFU counts after 4 and 8 weeks of treatment (*p* < 0.05, 4 weeks and *p* < 0.001, 8 weeks). It was not possible to conclude whether treatment induced differential culturability or failed to remove the DCB produced during infection.

### DMF eliminates Mtb DCB from the infected murine lungs

We previously hypothesized that the *in vivo* environment promoted the generation of DCB and Rpf-dependent mycobacteria and immunomodulation might interfere with this process (Turapov et al., 2014). We therefore treated mice with DMF, an approved drug for treatment of multiple sclerosis and psoriasis, which possesses anti-inflammatory and immunomodulatory properties (Kourakis et al., 2020). We determined that the minimum inhibitory concentration of DMF for the Mtb strain used in this study and grown in supplemented 7H9 medium is 25 µM; thus, DMF represents a drug with anti-inflammatory and anti-TB effects.

However, DMF treatment for 4 or 8 weeks had no effect on plateable Mtb (**Figures 2A, B**); the CFU counts from the control and DMF-treated mice did not differ significantly (*p* > 0.05). Importantly, the MPN and MPN_CSN counts were also similar to CFU counts obtained with DMF-treated mice (**Figures 2A, B**). The RI values for DMF-treated samples were below 0.2, compared with RIs of 1.73 and 1.83 for the control PEG-treated group at 4 and 8 weeks of treatment (**Figure 2C**). Hence, DMF treatment removed DCB Mtb from the infected lungs.

### Addition of DMF to standard regimen removes DCB from the lungs and spleens after 8 weeks of treatment

**Figure 3:**
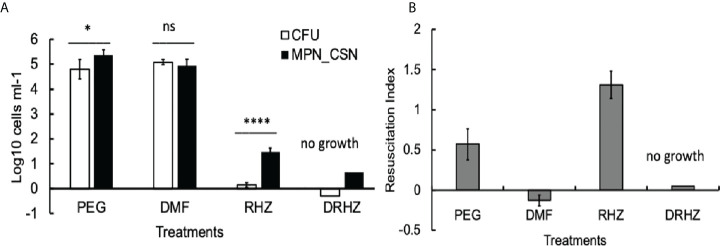
Effect of dimethyl fumarate (DMF), standard treatment and standard treatment with DMF on Mtb loads in the infected spleens after 8 weeks of treatment. **(A)** Spleen homogenates were used for CFU and MPN_CSN assays. Data shown as means ± STDV (*N* = 5) **(B)**. The resuscitation indices were calculated using the following formula: RI= log_10_ (MPN_CSN ml^-1^) – log_10_ (CFU ml^-1^). Ns, non-significant; *p* > 0.05; *p < 0.05; ****p < 0.0001 unpaired *t*-test. No Mtb were recovered in the DHZR group; the presented bars indicate the limit of detection for the MPN assay 4.6 cell ml^-1^ and 0.25 cell ml^-1^ (CFU). PEG, control PEG 400 group; DMF, dimethyl fumarate group; RHZ, rifampicin, isoniazid and pyrazinamide group; and DRHZ, rifampicin, isoniazid, pyrazinamide and dimethyl fumarate group.

Finally, we tested whether the addition of DMF to the RHZ regimen would impact on Mtb loads in the infected organs. After 4 weeks of treatment, all Mtb counts in the DRHZ and RHZ groups were very similar (CFU, MPN, and MPN_CSN), with an RI of 0.44, suggesting the presence of DCB (**Figures 2A, C**). However, 8-week treatment led to a reduction in MPN and MPN_GSN counts in the DHRZ group to 0.06, whereas the RI for RHZ lung samples was 0.43 (**Figure 2C**). These results suggest that the combined DRHZ treatment eliminated DCB from the lungs after 8 weeks. These findings were further confirmed by assessment of Mtb loads in the infected spleens after 8 weeks of treatment (**Figure 3A**). DCB_CSN_ were present in the control spleens; however, their proportion was lower as manifested by an RI of 0.5 compared with an RI of 1.83 in the lungs (**Figure 3B**). Interestingly, the RI value obtained for Mtb from the RHZ-treated spleens was 1.3, even though all Mtb counts were very low (**Figure 3B**). DMF completely eliminated all Mtb from the spleens because no Mtb were detected when the entire homogenates were used for assessment by CFU and MPN count assays. These findings suggest that while DMF, on its own, has no bactericidal effect on plateable Mtb, it eliminates DCB from infected organs.

## Discussion

The prolonged TB treatment creates substantial health, logistical and cost challenges (Dartois and Rubin, 2022). Recent drug trials have identified potential markers of patients who might be treated for shorter or longer periods (Imperial et al., 2018). However, our current understanding of Mtb persisters, the bacteria which are associated with failed treatment, is still limited. The Mtb persisters seemed to be represented by multiple populations, triggered by various factors (Gold and Nathan, 2017; Sarathy and Dartois, 2020). Importantly, some persisters are difficult to detect because they need special resuscitation media for cultivation. These DCB persisters are abundant in clinical samples and are more resistant to treatment. While animal studies have shown that DCB could be removed from the infected organs by increased concentrations of rifampicin (Hu et al., 2015) or bedaquiline (Hu et al., 2019) containing regimens, we still do not have a clear strategy for prevention of DCB formation or DCB removal from TB patients. The intrinsic challenge of DCB persisters is that the immune response directed to destroy Mtb may, in fact, create a specific environment that stimulates the generation of DCB. The excessive immune response results in lung damage and pathology, accompanied by the formation of lesions and cavities, the perfect environment for further persister formation and rise of drug-resistant Mtb (Sarathy and Dartois, 2020). Hence, finding drugs that control Mtb growth and reduce the immune response could provide a plausible solution for the Mtb persister challenge. Here, we present data demonstrating that application of anti-inflammatory drug DMF completely eliminates DCB from the infected lungs and spleens after 8 weeks of treatment. This pilot study was only designed for a relatively short course of infection, and further experiments are necessary to confirm whether the inclusion of DMF may reduce the duration of treatment and prevent TB relapse. It remains to be established whether DCB are particularly vulnerable to DMF or whether DMF breaks an ongoing cycle of DCB formation during the prolonged infection. We are currently testing the effect of DMF on DCB obtained *in vitro*. Future studies will reveal the molecular mechanisms of DMF-mediated elimination of DCB persisters from infected organism by identification of host signalling pathways, careful monitoring of cytokine levels in treated and untreated animals, investigation of metabolomics and application of other immunomodulatory chemicals such as vitamin D, steroid and non-steroid anti-inflammatory drugs (reviewed by Tobin, 2015). Design and synthesis of DMF derivatives with mycobactericidal effect may further improve the observed effects. Identification of individuals with high numbers of DCB using host proteomics (Beltran et al., 2020) or transcriptomics approaches (Singhania et al., 2018) will contribute to the development of patient-tailored TB therapy.

## Data availability statement

The raw data supporting the conclusions of this article will be made available by the authors, without undue reservation.

## Ethics statement

This study was reviewed and approved by Animal experiments were performed in accordance with the Animals (Scientific Procedures) Act, under project license P7B01C07A.The University of Leicester Ethical Committee and the U.K. Home Office approved the experimental protocols.

## Author contributions

GM, VM and DK conceived of and designed the experiments. SG, GM and VM secured funding. SG, OT and GM performed the experiments and analysed the data. All authors contributed to the article and approved the submitted version.

## Funding

This research was funded by the Medical Research Council Leicester Confidence in Concept 2019 award (MRC CiC 2019), grant number MC_PC_19043 (SG and GM), and the Russian Science Foundation under grant 21-15-00042 (VM).

## Acknowledgments

We thank Professor Andrea Cooper and Dr. John Pearl for the provision of Mtb strain and useful discussions. We acknowledge the Centre for Core Biotechnology Services at the University of Leicester for support with containment level 3 experiments. We are grateful to Jennifer Schofield for technical help with the experiments. We would like to acknowledge the staff at the Division of Biomedical Services, University of Leicester, for their care of the animals and their assistance in experimental procedures. DK thanks the Novo Nordisk Foundation for funding (grant NNF10CC1016517). The funders had no role in study design, data collection and analysis, decision to publish or preparation of the manuscript.

## Conflict of interest

Author DK was employed by The Novo Nordisk Foundation Centre for Biosustainability.

The remaining authors declare that the research was conducted in the absence of any commercial or financial relationships that could be construed as a potential conflict of interest.

## Publisher’s note

All claims expressed in this article are solely those of the authors and do not necessarily represent those of their affiliated organizations, or those of the publisher, the editors and the reviewers. Any product that may be evaluated in this article, or claim that may be made by its manufacturer, is not guaranteed or endorsed by the publisher.
